# Identification of the AHP family reveals their critical response to cytokinin regulation during adventitious root formation in apple rootstock

**DOI:** 10.3389/fpls.2024.1511713

**Published:** 2025-01-15

**Authors:** Ke Li, Huan Li, Wei Ling Liang, Jing Ju Liu, Hui Yue Tian, Li Hu Wang, Yan Hong Wei

**Affiliations:** ^1^ School of Landscape and Ecological Engineering, Hebei University of Engineering, Handan, Hebei, China; ^2^ Institute of Forestry and Fruit Science, Hebei University of Engineering, Handan, Hebei, China; ^3^ College of Horticulture, Yangling Subsidiary Center Project of the National Apple Improvement Center, Northwest Agriculture & Forestry University, Yangling, China

**Keywords:** apple, adventitous root, cytokinin, MdAHP, 35S::MdAHP3 transgenic plants

## Abstract

Adventitious root (AR) formation is a bottleneck for vegetative proliferation. In this study, 13 AHP genes (MdAHPs) were identified in the apple genome. Phylogenetic analysis grouped them into 3 clusters (I, II, III), with 4, 4, and 5 genes respectively. The 13 MdAHPs family members were named *MdAHP1* to *MdAHP13* by chromosome positions. The physicochemical properties, phylogenetic relationship, motifs, and elements of their proteins were also analyzed. The amino acid quantity varied from 60~189 aa, isoelectric point lay between 4.10 and 8.93, and there were 3~7 protein-conserving motifs. Excluding *MdAHP6*, other members’ promoter sequences behaved 2-4 CTK response elements. Additionally, the expression characteristics of MdAHPs family members at key stages of AR formation and in different tissues were also examined with exogenous 6-BA and Lov treatments. The results showed that *MdAHP3* might be a key member in AR formation. GUS staining indicated that the activity of the *MdAHP3* promoter was also significantly enhanced by CTK treatment. The protein interactions of MdAHP3/MdAHP1 and MdAHP3/MdAHP6 were verified. Compared with WT, *35S::MdAHP3* transgenic poplars inhibited AR formation. The above experimental results suggested that MdAHP3, as a key family member, interacts with MdAHP1 and MdAHP6 proteins to jointly mediate AR formation in apple rootstocks.

## Introduction

Apple (*Malus domestica* Borkh.) is one of the most commercially significant fruits worldwide. Currently, dwarfing apple rootstocks are extensively utilized, which is beneficial for fruit tree growth, such as enhancing tree resistance. Dwarfing dense planting is also an essential indicator of the development of the modern apple industry globally. Nevertheless, apple rootstock breeding programs have not been well established in China (Li et al., 2021). In production, apple dwarf rootstock breeding mainly employs grafting, cuttings, and tissue culture, the induction of ARs is the key to rootstock breeding (Shi et al., 2021). AR formation is an essential aspect of apple breeding. The emergence of ARs expands the root system of plants and enables plant and cell regeneration. They are widely used in plant cuttings and tissue culture. Therefore, biological research on AR formation in apple rootstocks is an important field of developmental biology. The molecular regulation mechanism of AR formation and the identification of key members of relevant candidate functional genes are of great theoretical significance for guiding the genetic improvement of AR formation and developing asexual rootstock breeding technology.

The plant’s root system comprises primary roots, lateral roots, and ARs. Primary roots are generated during embryogenesis. Lateral roots and ARs are initiated and developed by differentiated cells after the embryo stage. Lateral roots develop from existing roots, while ARs arise from tissues such as the stem or leaves of a plant (Li et al., 2019). AR formation can occur during normal plant development and can reproduce naturally through nutritional structures. The occurrence of ARs can also be the plant’s response to environmental and physiological stimuli, such as darkness, floods, and other mechanical injuries (Geiss et al., 2009; Bellini et al., 2014). There are two ways to generate ARs: one is the direct organogenesis of self-cells (such as the formation layer, cortex, pericycle or vascular bundle), which involves cell redifferentiation; the other is formed indirectly from the callus and requires external mechanical injury stimulation (Hartmann and Kester, 1990). In general, direct pathways mostly occur in species that are easy to root, while indirect pathways mostly occur in species that are difficult to root. AR formation is generally divided into three developmental stages: induction, germination, and elongation (Li et al., 2009). In *Arabidopsis thaliana*, ARs can occur from pericycle cells in the xylem of intact hypocotyls (Gutierrez et al., 2012; Sukumar et al., 2013). Explants of tobacco leaves produce roots in parenchyma cells near the vascular bundle (Harbinder and Dhaliwal, 2003). AR formation depends on numerous factors, such as genetic background, developmental stage, hormones, and other internal as well as external factors (Geiss et al., 2009; Costa et al., 2013). It is mainly determined by genotype and regulated by various endogenous hormones and environmental factors. Among them, cytokinin (CTK) and auxin (IAA) interact at the metabolic signaling and transport levels to regulate AR formation, and their effects are antagonistic (Aloni et al., 2006; Zhang et al., 2022; Chandler and Werr, 2015). Additionally, the ability of AR formation differed significantly among different apple rootstocks, and CTK significantly inhibited AR formation in apple rootstocks under histoponic conditions (Mao et al., 2019), suggesting that CTK plays a crucial role in AR formation in apple rootstocks.

Cytokinin (CTK) is achieved through a two-component signaling pathway that interacts synergistically with other hormones in regulating plant growth and development as well as plant responses to various stresses (Li et al., 2022). Cytokinins are also significant hormones that regulate the architecture of the root system. Exogenous cytokinin induces the elongation of root hairs, whereas lines overexpressing cytokinin oxidase display a short-hair phenotype (Monden et al., 2022). As one of the key phytohormones synthesized in the root, cytokinin (CTK) regulates many significant plant processes by controlling cell division and tissue differentiation (Riefler et al., 2006; Yan et al., 2008).

In the multistep two-component system, a histidine-containing phosphotransmitter (HPt) mediates the phosphotransfer from an activated membrane histidine kinase receptor to a response regulator in the nucleus. In *Arabidopsis thaliana*, 8 sensor histidine kinases (AHKs), 5 HPt proteins (AHPs), and 22 response regulators (ARRs) have been identified. A number of independent lines of evidence indicate that the phosphorelay from AHK through AHP to ARR is involved in plant signaling networks (Hutchison and Kieber, 2002; Hwang and Sheen, 2001). Among them, the AHP family plays an important role in the entire cytokine signaling process, which is now also reported in the model plants. The AHPs are a family of six related proteins, including five (*AHP1*-*AHP5)* that contain the conserved amino acids required for functioning as a histidine phosphotransfer protein (HPt), and one (*APHP1/AHP6*) that is considered a pseudo-AHP, as it lacks the conserved His residue that is the target of phosphorylation (Suzuki et al., 2000; Mahonen et al., 2006). In addition, *AHP1*, *AHP2*, and *AHP4* accumulate in the nucleus in response to cytokinin treatment (Hwang and Sheen, 2001; Yamada et al., 2014), indicating that the AHPs respond to cytokinin in plant cells. CTK signaling transduction, AHP2, AHP3, AHP5 of AHP in *Arabidopsis* were partially redundant positive regulators of CTK signal by reduced growth of the hypocotyl and root (Zhao et al., 2021). Additionally, recent studies have unveiled the crucial role of *AHP3* in governing root development in *Arabidopsis thaliana* (Hutchison et al., 2006; Wang et al., 2018). Hence, it is hypothesized that *MdAHP3* in apples might act as a key element in ARs formation. Nevertheless, additional exploration regarding the impact of *AHP3* on the root system in apples is requisite.

To date, the most direct evidence that HPts mediate cytokinin signaling in plants has come from experiments using cultured periwinkle cells. In these experiments, the cytokinin inducibility of a response regulator was reduced when a His phosphotransfer protein was silenced using RNA interference (Papon et al., 2004). Further evidence for the role of the AHPs has come from the observation that overexpression of *AHP2* results in a slight increase in sensitivity to cytokinin in root elongation assays (Suzuki et al., 2000). By contrast, recent genetic analysis indicates that *APHP1*/*AHP6*, a predicted pseudo-AHP, acts as a negative regulator of the cytokinin response pathway, most likely through a dominant negative mechanism. In general, a large number of studies have shown that AHPs play an important role in regulating plant growth and development. However, current work on the analysis and identification of the AHP family has mainly focused on model plant species such as *Arabidopsis* and *tomato* (Mahonen, 2006). The molecular mechanism of AHP family members in apples related to plant development is unclear, especially as the study of MdAHPs regulating AR formation in apple rootstocks has not been reported.

The molecular mechanism of AHP family members in apple involved in plant development is unclear, especially regarding the regulation of AR formation by MdAHPs in apple rootstocks. Currently, the mechanism of CTKs in AR formation is undefined, and the cellular mechanisms for root regeneration regulation are lacking. In this study, we systematically identified apple AHP family members, analyzed their evolutionary relationships, gene structure, properties, protein network prediction, and tissue expression. The expression patterns of MdAHPs in different tissues (root, xylem, stem, and leaf) and at critical AR formation stages in ‘M9-T337’ seedlings under exogenous treatment were verified. Additionally, a key member, *MdAHP3*, was selected and its function characterized. The results provide a theoretical basis for clarifying the MdAHPs family’s structure and functions, and for subsequent studies on AR formation and regulatory networks in apple rootstocks.

## Materials and method

### Identification and phylogenetic analysis of MdAHPs in apple (*Malus Domestica* Borkh.)

The amino acid sequences of AHPs in *Arabidopsis* were download from the information resource website (TAIR, https://www.arabidopsis.org/), and used as a query to search against the Genome Database for Rosaceae [apple genome (GDDH13 V1.1; https://www.rosaceae.org/] to predict candidate *MdAHPs* family members. Proteins with a non-significant E-value and those with incomplete or lacking domains were removed. The 13 MdAHP genes obtained were designated *MdAHP1* to *MdAHP13* based on their chromosomal locations, as in previous study (Xu et al., 2015; Cao et al., 2024; Jiang et al., 2024).

The protein sequences of *Arabidopsis* and apple AHPs were aligned by the ClustalW program with default parameters. The phylogenetic tree among the apple and *Arabidopsis* AHP proteins was constructed with the neighbor-joining method using the MEGA 6.0 program, with the parameter settings of ‘P-distance’, ‘Complete Deletion’, and 1000 bootstrap replicates. The physicochemical characteristics of proteins, such as isoelectric point, molecular weight, instability index, major amino acids, and aliphatic index, were predicted with the ExPASy program (http://web.expasy.org/protparam/) (Cao et al., 2024; Jiang et al., 2024).

### Gene structure, conserved motif, and promoter sequence analysis

Login to the online MEMES database (http://meme-suit.org/tools/meme), and download the conserved motifs of AHP proteins from this database. Set the number of motifs parameter to 10, and keep the rest of the parameters as default. Obtain the 1,500-bp genomic DNA sequence upstream of the start codon (ATG) of each MdAHPs gene from the apple genome sequence. Identify the *cis*-elements in the promoters using the PlantCARE database (http://bioinformatics.psb.ugent.be/webtools/plantcare/html/).

### Genes expression characterization analysis in different genotypes of apple and protein function linkage network prediction

The gene expression of different tissues (flowers, fruits, seedlings, seeds, leaves, roots, and stems) of 10 different genotypes (M14, M20, M49, M67, M74, GD, X4102, X8877, X442×X2596, and X3069×X922) was downloaded from GEO data (http://www.ncbi.nlm.nih.gov/geo/; login number: GSE42873), and the expression heat map was plotted using HEML1.0 software. The interaction networks of 13 MdAHP proteins were analyzed using protein patterns with high specificity from the String Protein Interaction Database (http://string-db.org/), and the species parameters were selected for *A. thaliana*.

### Plant materials and treatments

Samples were collected from ‘M9-T337′ apple rootstock plantlets grown in tissue culture at the Hebei University of Engineering, Handan, China. The morphologically uniform cuttings were maintained under 16 h of light at 25 ± 1°C, followed by 8 h of dark at 15 ± 1°C. The stem cuttings were divided into three groups. The first group of morphologically uniform micro-cuttings were treated with indole-3-butyric acid (IBA), which is widely used to promote AR formation. The rooting medium was composed of 1/2 MS, 1 mg.L^− 1^ IBA, 25 g.L^−1^ sugar, 7.5 g.L^−1^ agar, and pH 5.8; it was named control. The second group of morphologically uniform micro-cuttings were treated with IBA and 6-BA, the medium was composed of 1/2 MS, 1 mg.L^−1^ IBA, 0.5 mg.L^−1^ 6-BA, 25 g.L^−1^ sugar, 7.5 g.L^−1^ agar, and pH 5.8; it was named IBA+ 6-BA treatment. The third group of morphologically uniform micro-cuttings were treated with IBA and Lovastatin (CTK synthetic inhibitor); the medium was composed of 1/2 MS, 1 mg.L^−1^ IBA, 0.5 mg.L^−1^ lovastatin, 25 g.L^−1^ sugar, 7.5 g.L^−1^ agar, and pH 5.8; it was named IBA+Lov treatment.

In the current study, the sampling time points of the samples were set as follows: 1, 3, 7, 11, and 16 d. Sixty randomly selected plants at each time point were similar in growth. The stem cuttings were sampled, and three biological replicates were set at each sampling time point. The sampling site was 0.3 - 0.5 cm at the base of the stem. Additionally, different tissue parts (side roots, stems, leaves, flowers, fruits, flower buds, and axillary buds) of ‘M9-T337’ apple rootstock were also collected in the germplasm resource garden of the ‘Yangling Branch of the National Apple Improvement Center’ for subsequent tissue-specific expression analysis. After being quickly frozen in liquid nitrogen, all the above samples were stored at − 80°C until use.

### Extraction of plant DNA and RNA, cDNA synthesis, and quantitative real-time PCR

RNA was extracted using the CTAB method (Li et al., 2021; Yan et al., 2022). DNA was extracted using ‘M9-T337’ apple rootstock tissue culture seedling leaves. 1.2% agarose gel electrophoresis (200 V, 400 mA, 80 W, 15 min) and a micro-UV spectrophotometer (Thermo Nano Drop 2000, USA) were used to detect the quality of the extracted RNA and DNA. One microgram of total RNA was used as the template for first-strand cDNA synthesis, using the PrimeScript RT Reagent kit (Takara, Shiga, Japan) following the manufacturer’s instructions.

The expression patterns of *MdAHP1* to *MdAHP13* were examined by RT-qPCR. Primer pairs for quantitative real-time PCR (RT-qPCR) were designed using Primer Premier 6.0 (Premier Biosoft, Palo Alto, CA, USA) (**Supplementary Table 1**). RT-qPCR was conducted as described in previous research (Jia et al., 2020; Xing et al., 2015). An apple ACTIN gene was used for normalization. Each samples consisted of three biological and technical replicates. The 2^− ΔΔ^Ct method was used to calculate the relative expression levels (Livak and Schmittgen, 2001).

### Plasmid reconstruction and genetic transformation

The full-length cDNA of *MdAHP3* was cloned into the ‘M9-T337’ and pCAMBIA2300 vectors to construct overexpression vectors, which were then driven under the CaMV 35S promoter. Additionally, a 1.5-kb promoter fragment of the *ProMdAHP3::GUS* vector was amplified and inserted into the pCAMBIA1381 vector containing the GUS reporter gene.

To obtain transgenic poplars, the *35S::MdAHP3-GFP* construct vectors were transformed into the GV3101 strain and subsequently introduced into *poplar* (*Populus tomentosa* Carrière) using the A. tumefaciens-mediated leaf disk method (Shikakura et al., 2022). Transgenic lines were screened with 100 mg.L^−1^ kanamycin. The *pBI121–35S::GUS* and *ProMdAHP3::GUS* fusion constructs were instantaneously transformed into apple leaves that had grown for 5 weeks and were cultivated in the shade for 48 h. Different combinations of apple leaves were treated with water (as the control) and 6-BA (100 μmol.L^−1^). 5-Bromo-4-chloro-3-indolyl-β-glu-curonide (X-Gluc) was employed as a substrate for the GUS staining observation test (Shikakura et al., 2022).

### Yeast two-hybrid screening and confirmation

Y2H studies were carried out in accordance with the Yeast maker Yeast Transformation System 2 protocol (Clontech, Palo Alto, CA, USA). The pGAD424-MdAHP1 and pGBT9-MdAHP3 plasmids were co-transformed into Y2H Gold (Clontech, Palo Alto, CA, USA), and were then grown on the selection medium supplemented with SD base/-L/-T (SD base/-Leu/-Trp), followed by the medium supplemented with SD base/-L/-T/-H/-A (SD base/-Leu/-Trp/-His/-Ade), with or without 5-bromo-4-chloro-3-indolyl-α-d-galactopyranoside (X-α-Gal) to determine any interactions between *MdAHP1* and *MdAHP3*. The primers utilized for vector construction are listed in **Supplementary Table 1.**

### Bimolecular fuorescence complementation assay

The full-length coding sequences of MdAHP1 and MdAHP3 were respectively inserted into the 35S::pSPYNE-nYFP and 35S::pSPYCE-cYFP vectors. Subsequently, the resultant constructs were transformed into the Agrobacterium strain GV3101. Thereafter, different combinations were mixed and transformed into Nicotiana benthamiana leaves and cultured at 23°C for 48 h. YFP fluorescence was detected by a confocal laser-scanning microscope with excitation at 488 nm (Zeiss LSM 510 Meta, Jena, Germany). The primers utilized for BiFC are listed in **Supplementary Table 1.**

### Statistical analysis

Data were subjected to analysis of variance, and the means were compared using Student’s t-test at the 5% significance level. The SPSS 11.5 software (SPSS, Chicago, IL, USA) was employed for data processing. Figures were generated using SigmaPlot 10.0 (Systat Software, Inc.).

## Results

### Genome-wide identification of arabidopsis and apple *MdAHP* genes

**Figure 1:**
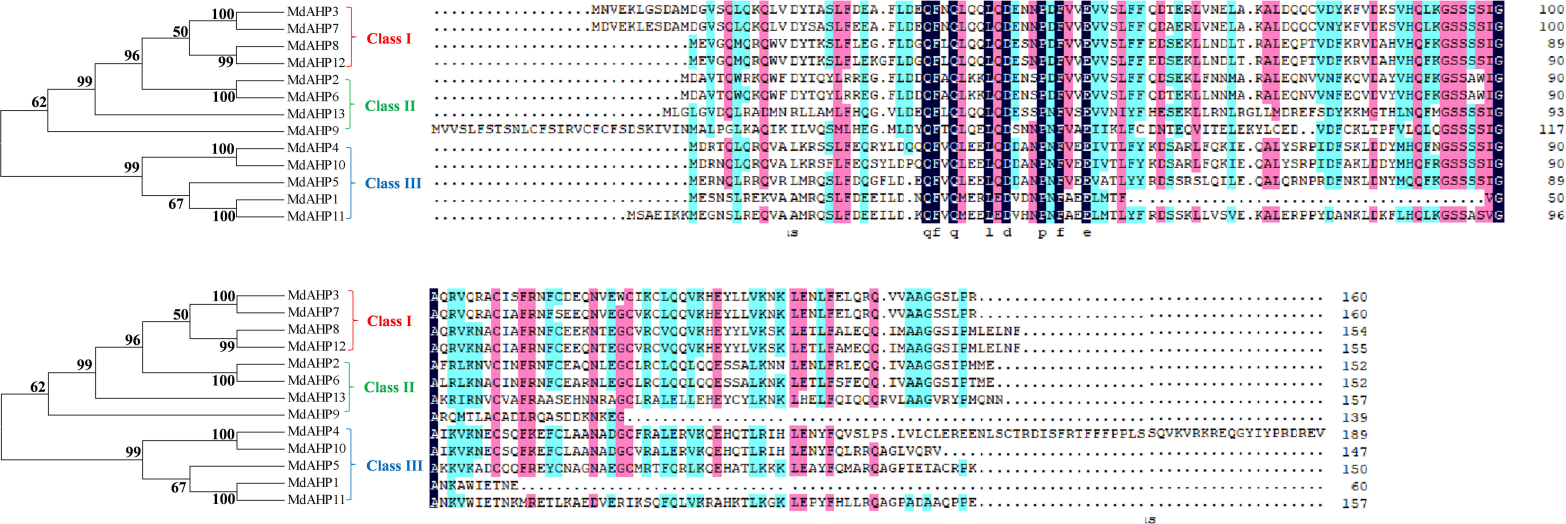
Multiple sequence alignment of MdAHPs proteins.

Eight AHP genes were previously identified and reported in the *Arabidopsis thaliana* (*A. thaliana*) genome, namely *AHP1*, *AHP2/AHP2-1*, *AHP3*, *AHP4*, *AHP5*, *AHP6*, and *DAHP2*. In this study, to elucidate the AHP genes in apple, we took the protein sequences of the eight AtAHP members in *A. thaliana* as a query basis to search for apple genome family members via BLASTP. After manual inspection and screening for confirmation with the NCBI conserved domain database, 13 candidate *MdAHP* genes were obtained (**Table 1**). The 13 *MdAHP* genes were situated on 12 chromosomes in the apple genome. Among them, chromosome 15 encompassed two genes, while chromosomes 2, 3, 4, 8, 9, 11, 12, 13, 14, 16, and 17 each harbored a single gene (**Table 1**). Additionally, multiple sequence alignment indicated that the majority of the MdAHP proteins shared the common four conserved domains (**Figure 1**). Among them, the MdAHP4 protein sequence was the longest, containing 189 amino acids; the MdAHP1 protein sequence was the shortest, consisting of 60 amino acids; the protein sequences of other MdAHPs family members were of lengths ranging between those of MdAHP1 and MdAHP4 (**Figure 1**).

**Table 1:** Characteristics of the AHPs gene families in *Arabidopsis thaliana* and Apple. Chr, Chromosome; CDS, Coding Sequence.

Name	Gene ID	Location	CDS (bp)	Peptide (aa)
AHP1	AT3G21510	Chr3:7578432-7579537	465	154
AHP2	AT3G29350	Chr3:11264379-11265408	471	156
AHP3	AT5G39340	Chr5:15748941-15750248	468	155
AHP4	AT3G16360	Chr3:5554351-5555518	438	145
AHP5	AT1G03430	Chr1:848159-849235	474	157
AHP6	AT1G80100	Chr1:30133818-30134652	465	154
AHP2	AT1G13330	Chr1:4568008-4569410	681	226
DAHP2	AT4G33510	Chr4:16116496-16118549	1524	507
MdAHP1	MD02G1065100	Chr02:5274203-5275042	219	60
MdAHP2	MD03G1272900	Chr03:5623827-35626300	459	152
MdAHP3	MD04G1212100	Chr04:29654492-29656643	483	160
MdAHP4	MD08G1111300	Chr08:9779829-9781444	573	189
MdAHP5	MD09G1203100	Chr09:18975574-18977109	453	150
MdAHP6	MD11G1293900	Chr11:41305280-41307736	459	152
MdAHP7	MD12G1226800	Chr12:30252651-30254431	483	160
MdAHP8	MD13G1176300	Chr13:14595883-14597631	465	154
MdAHP9	MD14G1019100	Chr14:1804629-1805852	420	139
MdAHP10	MD15G1090700	Chr15:6287020-6289085	444	147
MdAHP11	MD15G1195900	Chr15:15559650-15560922	474	157
MdAHP12	MD16G1178100	Chr16:15212549-15214510	468	155
MdAHP13	MD17G1211900	Chr17:25911289-25912804	474	157

Chr, Chromosome; CDS, Coding Sequence.

### The *MdAHP* family genes characterization analysis

The protein characteristics of MdAHPs were analyzed by ExPASy portal, encompassing molecular weight, isoelectric point, grand average of hydropathicity, instability index, major amino acid content, and aliphatic index (**Table 2**). The molecular weights of the analyzed MdAHP proteins ranged from 7,024.79 Da (MdAHP1) to 18,339.70 Da (MdAHP3). The molecular weights of the MdAHP proteins were less than 13,000 Da, indicating that MdAHP constitutes a group of micro-molecule proteins (**Table 2**). The isoelectric points (pI) of the MdAHP proteins ranged from 4.10 (MdAHP1) to 8.93 (MdAHP5), with MdAHP5 and MdAHP13 proteins being basic and the remainder being acidic (**Table 2**). The instability index values of the MdAHP proteins ranged from 28.96 (MdAHP8) to 61.14 (MdAHP10). Among them, MdAHP1, MdAHP4, MdAHP5, MdAHP9, MdAHP10, MdAHP11, and MdAHP13 exceeded 40, and all the aforesaid proteins were regarded as unstable (**Table 2**). The aliphatic index (AI) of the MdAHP proteins ranged from 59.27 (MdAHP5) to 96.76 (MdAHP9). Additionally, the hydrophilic index (GRA) of the MdAHP proteins ranged from 0.096 (MdAHP9) to 0.946 (MdAHP5), with the MdAHP9 protein being the least hydrophilic and the MdAHP5 protein being the most hydrophilic (**Table 2**).

**Table 2:** Amino acid compositions as well as physical and chemical characteristics of MdAHP proteins.

Name	Gene Locus^b^	GRAVY	CDS^d^ (bp)	e(aa)	MW	PI^i^	II^j^	AI^k^
**MdAHP1**	**MD02G1065100**	**-0.598**	**219**	**60**	**7024.79**	**4.10**	**46.18**	**73.17**
**MdAHP2**	**MD03G1272900**	**-0.392**	**459**	**152**	**17788.18**	**5.21**	**34.67**	**79.54**
**MdAHP3**	**MD04G1212100**	**-0.359**	**483**	**160**	**18339.70**	**4.74**	**32.74**	**90.06**
**MdAHP4**	**MD08G1111300**	**-0.581**	**573**	**189**	**22511.53**	**6.83**	**55.72**	**79.00**
**MdAHP5**	**MD09G1203100**	**-0.946**	**453**	**150**	**17613.88**	**8.93**	**56.17**	**59.27**
**MdAHP6**	**MD11G1293900**	**-0.359**	**459**	**152**	**17671.00**	**4.91**	**39.17**	**82.11**
**MdAHP7**	**MD12G1226800**	**-0.369**	**483**	**160**	**18250.54**	**4.72**	**39.71**	**90.69**
**MdAHP8**	**MD13G1176300**	**-0.256**	**465**	**154**	**17691.13**	**4.95**	**28.96**	**84.74**
**MdAHP9**	**MD14G1019100**	**0.096**	**420**	**139**	**15602.02**	**4.64**	**42.47**	**96.76**
**MdAHP10**	**MD15G1090700**	**-0.539**	**444**	**147**	**17323.66**	**6.83**	**61.14**	**83.61**
**MdAHP11**	**MD15G1195900**	**-0.657**	**474**	**157**	**18059.67**	**6.12**	**52.64**	**80.19**
**MdAHP12**	**MD16G1178100**	**-0.290**	**468**	**155**	**17837.30**	**4.95**	**29.54**	**81.68**
**MdAHP13**	**MD17G1211900**	**-0.343**	**474**	**157**	**18187.89**	**7.79**	**44.10**	**92.55**

GRAVY, grand average of hydropathicity; e (aa), amino acid number; MW, molecular weight, Da; PI, isoelectric point; II, Instability Index; AI, Aliphatic Index.

### Phylogenetic relationships and structure analysis among *MdAHP* genes

**Figure 2:**
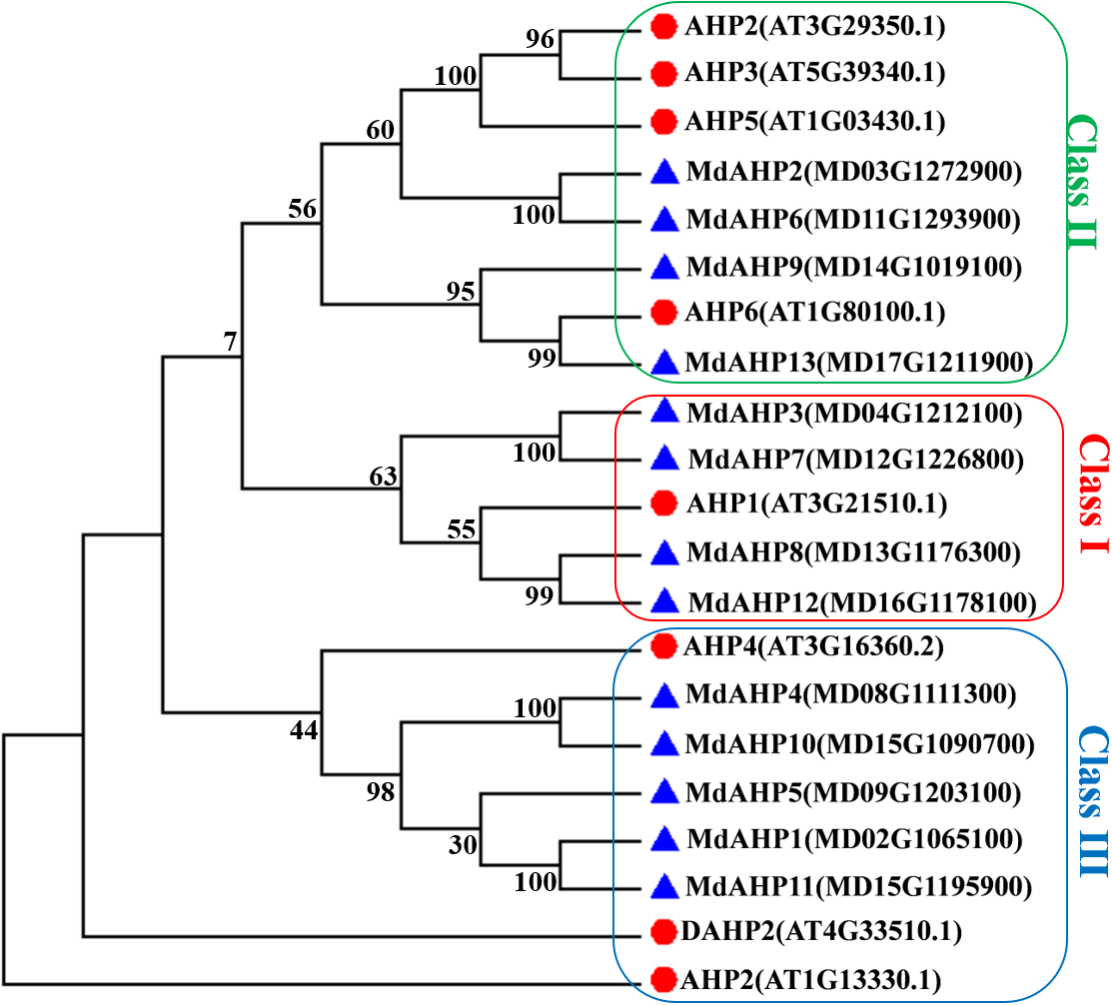
Phylogenetic analysis of the proteins in apple and *Arabidopsis* AHPs. Triangles and circles respectively indicate the proteins of apple and *Arabidopsis*.

To elucidate the evolutionary relationships among MdAHP proteins, a phylogenetic tree was constructed using 13 MdAHP proteins identified from apple and 8 AtAHP protein sequences from *Arabidopsis* (**Figure 2**). Based on the phylogenetic tree, the protein sequences were categorized into three groups: class I, class II, and class III. Class I comprised 5 proteins, class II contained 8 proteins, and class III incorporated 8 proteins (**Figure 2**). Among them, *MdAHP3*, *MdAHP7*, *MdAHP8* and *MdAHP12* presented higher similarity to *AHP1* in *Arabidopsis*; *MdAHP2* and *MdAHP6* in apple exhibited higher similarity with *AHP2*, *AHP3*, *AHP5* in *Arabidopsis*; *MdAHP9* and *MdAHP13* in apple displayed higher similarity to *AHP6* in *Arabidopsis*; *MdAHP1*, *MdAHP4*, *MdAHP5*, *MdAHP10* and *MdAHP11* manifested higher similarity with *AHP4* in *Arabidopsis* (**Figure 2**). The higher similarity among these genes implies that they might have similar functions.

**Figure 3:**
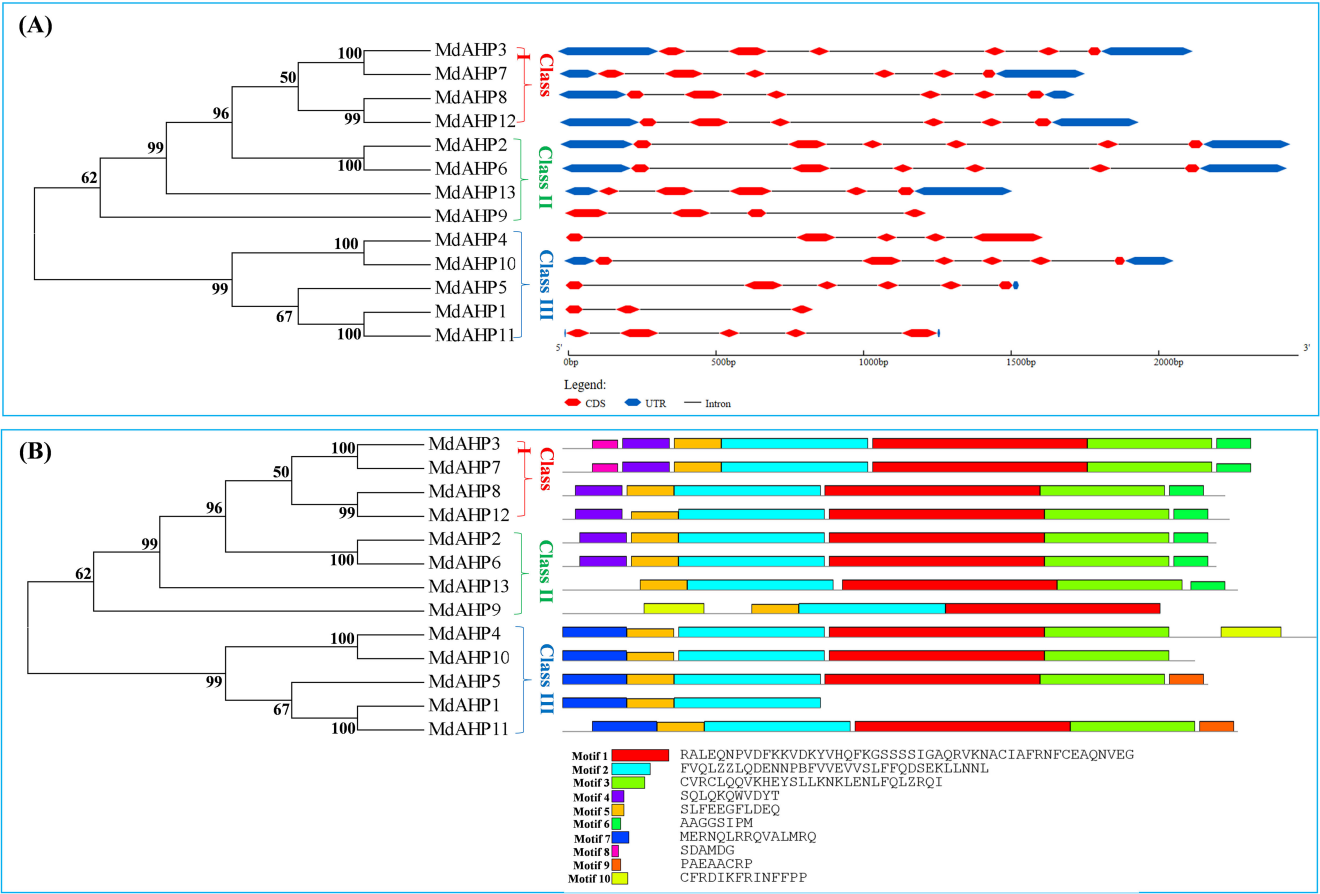
Analysis of the *MdAHP* gene structure. **(A)** An unrooted neighbor-joining tree was constructed based on the sequences of MdAHP proteins and the analysis of exon–intron composition. **(B)** the presented results are those of the protein motif analysis of MdAHP family members.

The Gene Structure Display Server was employed to exhibit the exon–intron structure based on the annotated apple genome. All the members of the MdAHP family contained 2 to 5 introns. The number and distribution of introns for the MdAHP genes were rather conserved within each class (**Figure 3A**). For instance, Class I, which included *MdAHP3*, *MdAHP7*, *MdAHP8*, and *MdAHP12*, was highly conserved and consisted of five introns and six exons (**Figure 3A**). Nevertheless, despite the genes *MdAHP1* and *MdAHP11* demonstrating high similarity in protein sequences, the distribution and location of exons were distinct (**Figure 3A**). These disparities suggested that the two genes may had functionally diverged during evolution. However, differences in gene structure, apart from protein sequences, may not simply be equivalent to functional divergence, still need further study.

Conserved motif analysis disclosed that the quantity of conserved motifs of the 13 MdAHP genes in apple varied from 3 to 7. There were only 3 conserved motifs for *MdAHP1*. *MdAHP3* and *MdAHP7* possessed 7 conserved motifs, whereas the rest of the MdAHP members had between 4 and 6 conserved motifs. (**Figure 3B**).

### Analysis of the expression pattern of *MdAHPs* in different of tissue and genotypes apple

**Figure 4:**
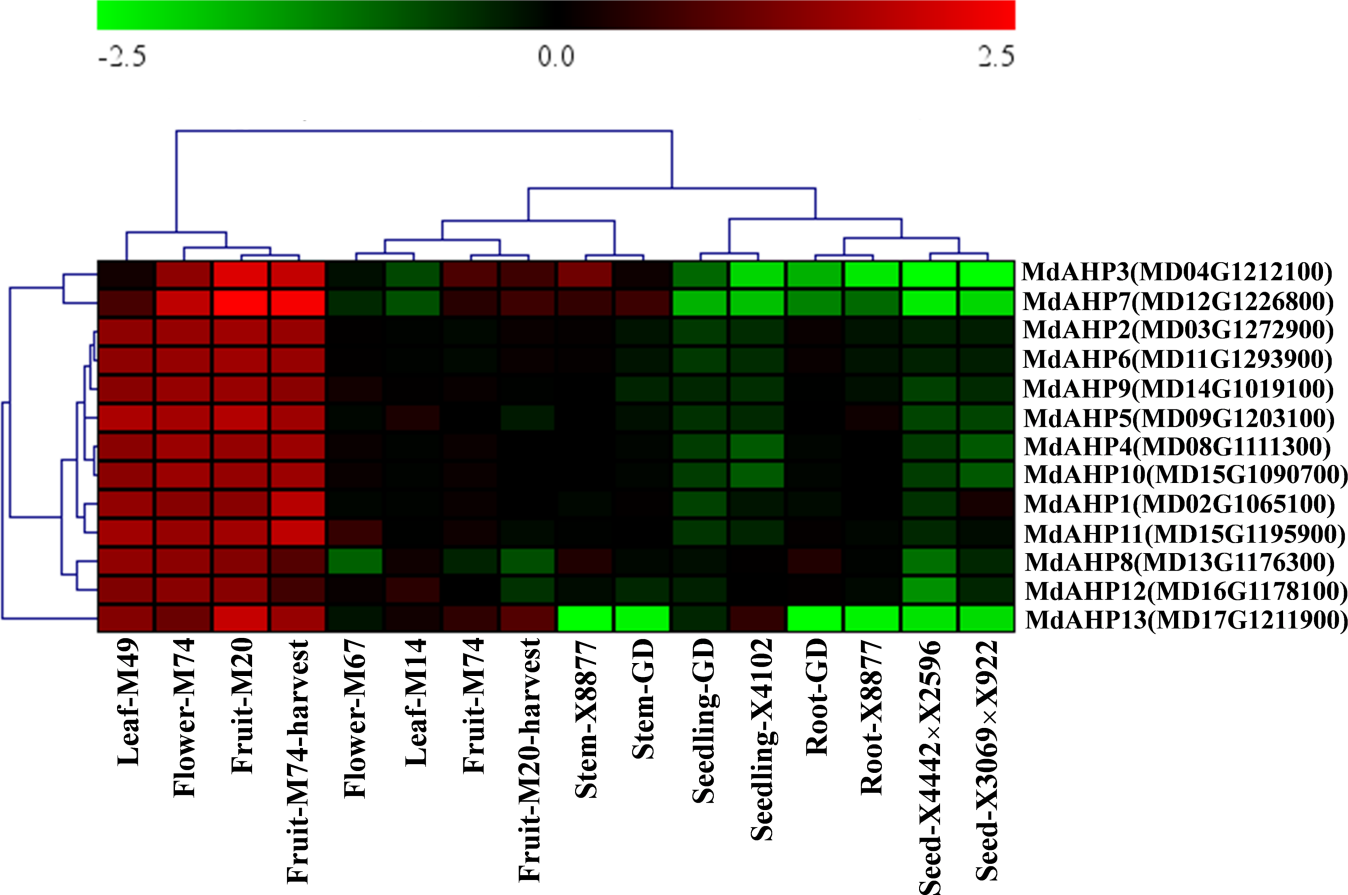
The expression pattern of the MdAHP gene in different organs of apple varieties. The different organs encompass leaf, flower, fruit, stem, seedling, root, and seed; the apple varieties comprise M49, M74, M20, M67, M14, X8877, GD, X4102, X8877, X4442×X2596, and X3069×X922.

To initially validate the prediction results and explore the expression of the MdAHPs gene family in different organs, the gene expression patterns of different tissues (flowers, fruits, seedlings, seeds, leaves, roots and stems) from ten different hybrids (M14, M20, M49, M67, M74, GD, X8877, X4102, X4442×X2596 and X3069×X922) are downloaded from the GEO database. The expression of the MdAHP family genes in different tissues is shown in **Figure 4**, and 13 MdAHP genes are found to be differentially expressed in different tissues. It can be found from the results that all 13 MdAHP family genes show relatively low expression in GD seedlings, X4102 seedlings, GD and X8877 roots, X4442X2596 seeds, and X3069X922 seeds, while they show relatively high expression in M49 leaves, M74 flowers, M20 fruits and M74 harvest fruits (**Figure 4**). In addition, their expression is between the former two groups in M67 flowers, M14 leaves, M74 fruits, M20 harvest fruits, X8877 stems and GD stems (**Figure 4**). Based on the above results, it is known that *MdAHP3*, *MdAHP7* and *MdAHP13* show the most significant differences in expression in different varieties and tissues (**Figure 4**). This also indicates that these genes may have potential functions in regulating the development of different organs. These results also provide a basis for further studies on the functional analysis of MdAHP genes.

### Effect of phytohormone treatments on *MdAHP* expression during AR formation

**Figure 5:**
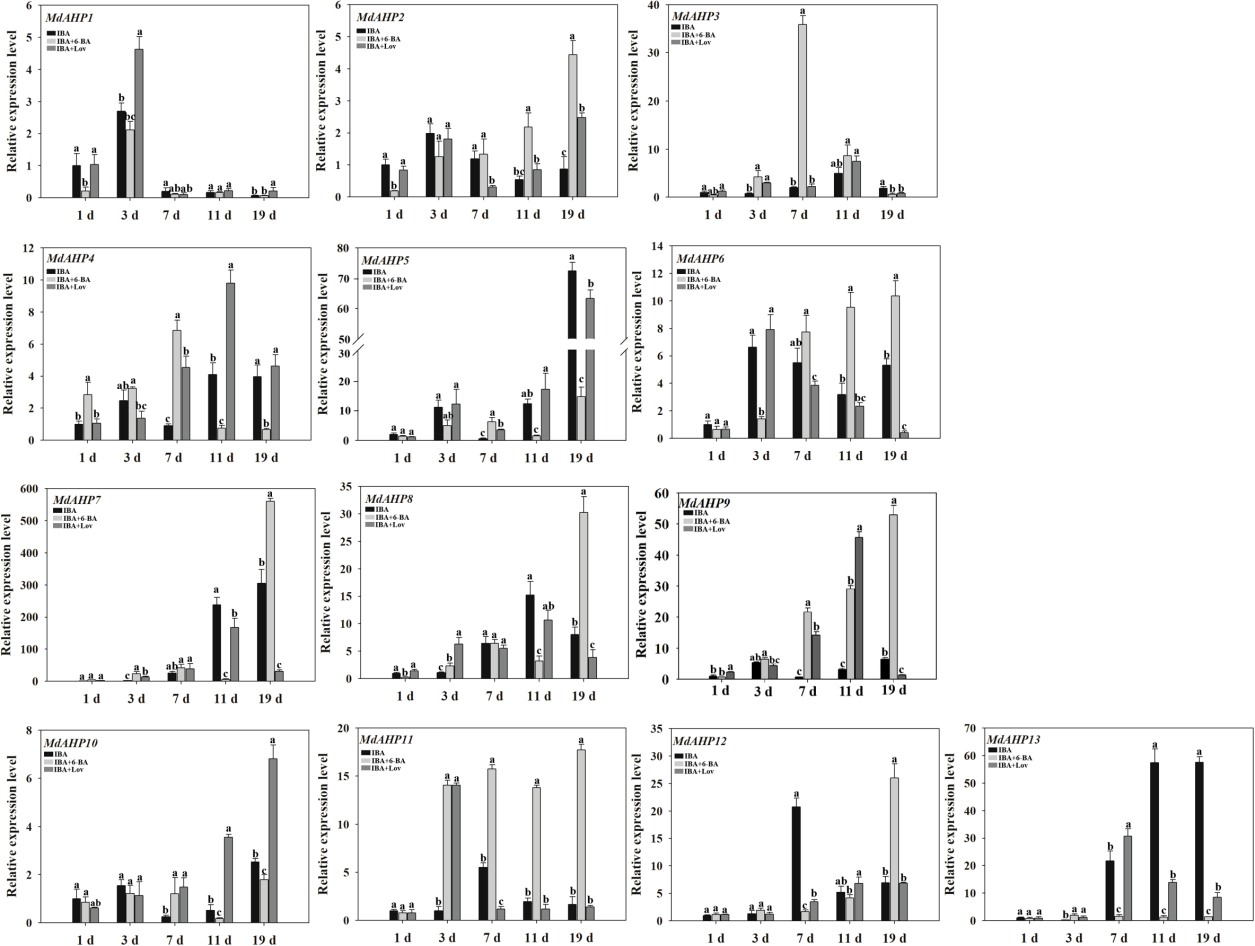
*MdAHP* gene expression profiles during the key stages of AR formation. The expression profiles of the MdAHP family genes as determined by RT-qPCR. Samples were collected at 1, 3, 7, 11, and 19 days during AR formation. Each value represents the mean ± standard error of three replicates. Letters a–c denote a significant difference at the 0.05 level.

To assess the potential effects of cytokinins on MdAHP expression during AR formation in apple, the expression patterns of each gene were measured at the stages of 1 d, 3 d, 7 d, 11 d, and 19 d in “M9-T337” apple rootstock seedlings through IBA, IBA + 6-BA, and IBA + Lov treatments respectively. The sole IBA treatment promoted ARs formation, while the treatment of IBA and 6-BA inhibited ARs formation. In addition, Lovastatin (Lov) as a cytokinin synthesis inhibitor, when combined with IBA, could further promote the ARs formation. Through transcriptome data analysis, MdAHPs family members were screened, and their expression patterns were analyzed by heatmap clustering (**Supplementary Figure 1**). Furthermore, the expression characteristics of MdAHPs family gene members were analyzed by RT-qPCR (**Figure 5**). In terms of the general expression trends, they can be classified into five categories. Category 1: Under all three treatments, the expression of *MdAHP1* peaked at 3 d. Additionally, compared with IBA and IBA + 6-BA treatments, it was significantly down-regulated at 1 d and 3 d by IBA + 6-BA treatment (**Figure 5**). Category 2: under the three treatments, the expression levels of *MdAHP2*, *MdAHP5*, *MdAHP8*, and *MdAHP10* were low at stages of 1 d, 3 d, 7 d and 11 d; while the expression levels of *MdAHP2* and *MdAHP8* under IBA+6-BA treatment was significantly higher than that of IBA and IBA+Lov treatments at 19 d; the expression levels of *MdAHP5* and *MdAHP10* under IBA+6-BA treatment were significantly lower than that of IBA+Lov treatment at 19 d (**Figure 5**). Category 3: Compared with IBA and IBA + Lov treatments, the expression levels of *MdAHP3* and *MdAHP4* were significantly up-regulated at 7 d by IBA + 6-BA treatment (**Figure 5**). Category 4: Under IBA + 6-BA treatment, *MdAHP6*, *MdAHP7*, and *MdAH12* exhibited the highest expression levels at 19 d during AR formation (**Figure 5**). Category 5: Under IBA + 6-BA treatment, the relative expression level of *MdAHP11* was higher than that of IBA and IBA + Lov treatments at 3 d, 7 d, 11 d, and 19 d. However, no significant difference was observed at 1 d among IBA, IBA + 6-BA, and IBA + Lov treatments (**Figure 5**). Although the exact fold change of the DEGs varied between RNA-Seq and RT-qPCR, the expression trends of DEGs detected by RNA-sequencing and RT-qPCR were largely consistent (**Figure 5**; **Supplementary Figure 1**).

### *MdAHP* expression patterns of different tissues in ‘M9-T337’ apple rootstock

**Figure 6:**
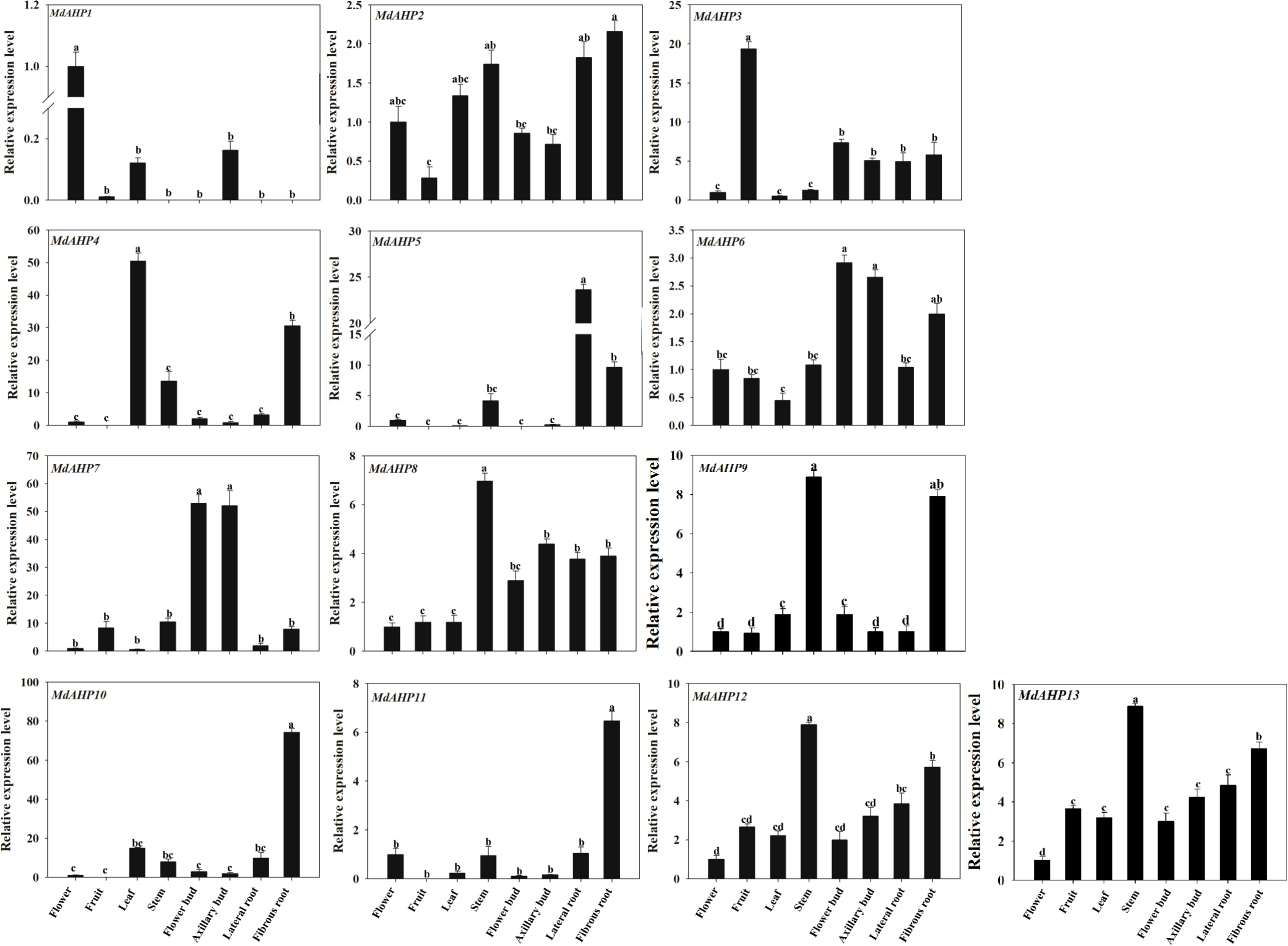
The expression patterns of the MdAHP family genes in various tissues (flower, fruit, leaf, stem, flower bud, axillary bud, lateral bud, fibrous bud) of the ‘M9-T337’ apple rootstocks. Each value represents the mean ± standard error of three replicates. Letters a–c indicate a significant difference at the 0.05 level.

The expression patterns of *MdAHP1* to *MdAHP13* in fruits, flowers, leaves, stems, flower buds, axillary buds, lateral roots, and fibrous roots were determined in “M9-T337” apple rootstocks and can be broadly classified into eight categories. Category 1: The relative expression level of *MdAHP1* was higher in flowers than in other tissues, and it was scarcely expressed in lateral roots, stems, and fibrous roots (**Figure 6**). Category 2: The relative expression level of *MdAHP3* was highest in fruits and lowest in leaves and flowers (**Figure 6**). Category 3: *MdAHP4* was expressed at the highest level in leaves, followed by fibrous roots, and not in fruits (**Figure 6**). Category 4: *MdAHP8* and *MdAHP12* were highly expressed in stems, followed by lateral and fibrous roots, and had relatively low expression in other tissues (**Figure 6**). Category 5: The relative expression levels of *MdAHP6* and *MdAHP7* in flower buds and axillary buds were significantly higher than in other tissue sites (**Figure 6**). Category 6: *MdAHP5* was expressed at the highest level in lateral roots, followed by fibrous roots, and not in fruits and flower buds (**Figure 6**). Category 7: The relative expression levels of *MdAHP10* and *MdAHP11* in fibrous roots were significantly higher than in other tissue sites (**Figure 6**). Category 8: *MdAHP2* was highly expressed in stems, lateral roots, and fibrous roots, and was lowly expressed in fruits, flower buds, and axillary buds (**Figure 6**).

### Analysis of promoter elements of *MdAHP* gene family members

To further investigate the regulatory mechanisms and potential functions of MdAHP genes, *cis*-element motifs associated with responses to environmental factors and phytohormones were detected in the 1.5-kb promoter region upstream of the start codon (ATG) (**Table 3**). Light signaling and stress response elements were identified. Additionally, hormones-related *cis*-acting elements, including those related to auxin, zeatin, gibberellin, ethylene, methyl jasmonate, salicylic acid, and abscisic acid, were also identified in **Table 3**. The number of *cis*-acting elements among the 10 identified in the MdAHP family genes ranged from 11 *(MdAHP10*) to 27 (*MdAHP6* and *MdAHP11*). Since this study is based on the cytokines signaling pathway, we focused on the number of zeatin metabolism-elements on the promoter sequences of AHP family genes. From the results, we can indicate that *MdAHP3* and *MdAHP5* contain 4 zeatin metabolism-elements each (**Table 3**). However, no zeatin metabolism-element in *MdAHP6* promoter sequence was observed (**Table 3**). The other MdAHP family members have zeatin metabolism-elements ranging from 0 to 4 (**Table 3**). The presence of regulatory elements in the MdAHP genes indicates that its family members are affected not only by the external environment (e.g. light, cold, drought, etc.) but also by various hormones (cytokines, jasmonic acid, auxin, gibberellin, ethylene, etc.). Thus, we presume that the MdAHP family genes can be involved in regulating the development of apple by responding to these signaling factors.

**Table 3:** Predicted cis-elements in the MdAHPs promoters.

Gene name	MeJA	Stress	Gibberellin	Salicylic acid	Auxin	Abscisic acid	Meristem	Ethylene	Circadian	Zeatin metabolism	Total
*MdAHP1*	2	1	0	1	0	4	0	0	8	2	18
*MdAHP2*	4	0	0	0	0	2	0	0	12	1	19
*MdAHP3*	0	0	0	2	1	4	0	0	15	4	26
*MdAHP4*	0	0	0	3	0	2	0	0	9	4	18
*MdAHP5*	4	1	0	0	0	2	1	0	4	2	14
*MdAHP6*	4	1	0	0	1	5	0	0	16	0	27
*MdAHP7*	4	1	0	2	0	0	1	0	7	3	18
*MdAHP8*	2	1	1	1	0	2	0	0	8	2	17
*MdAHP9*	0	3	0	0	1	0	1	0	9	2	16
*MdAHP10*	0	3	0	0	0	1	1	0	5	1	11
*MdAHP11*	4	4	0	1	0	3	0	0	12	3	27
*MdAHP12*	2	3	1	0	1	1	0	0	6	2	16
*MdAHP13*	0	2	1	1	1	0	0	0	7	2	14

The 1.5 kb sequence upstream from the start codon of MdAHPs genes were analyzed using the PlantCARE database.

### Cytokines can enhance the expression activity of *MdAHP3* promoter

**Figure 7:**
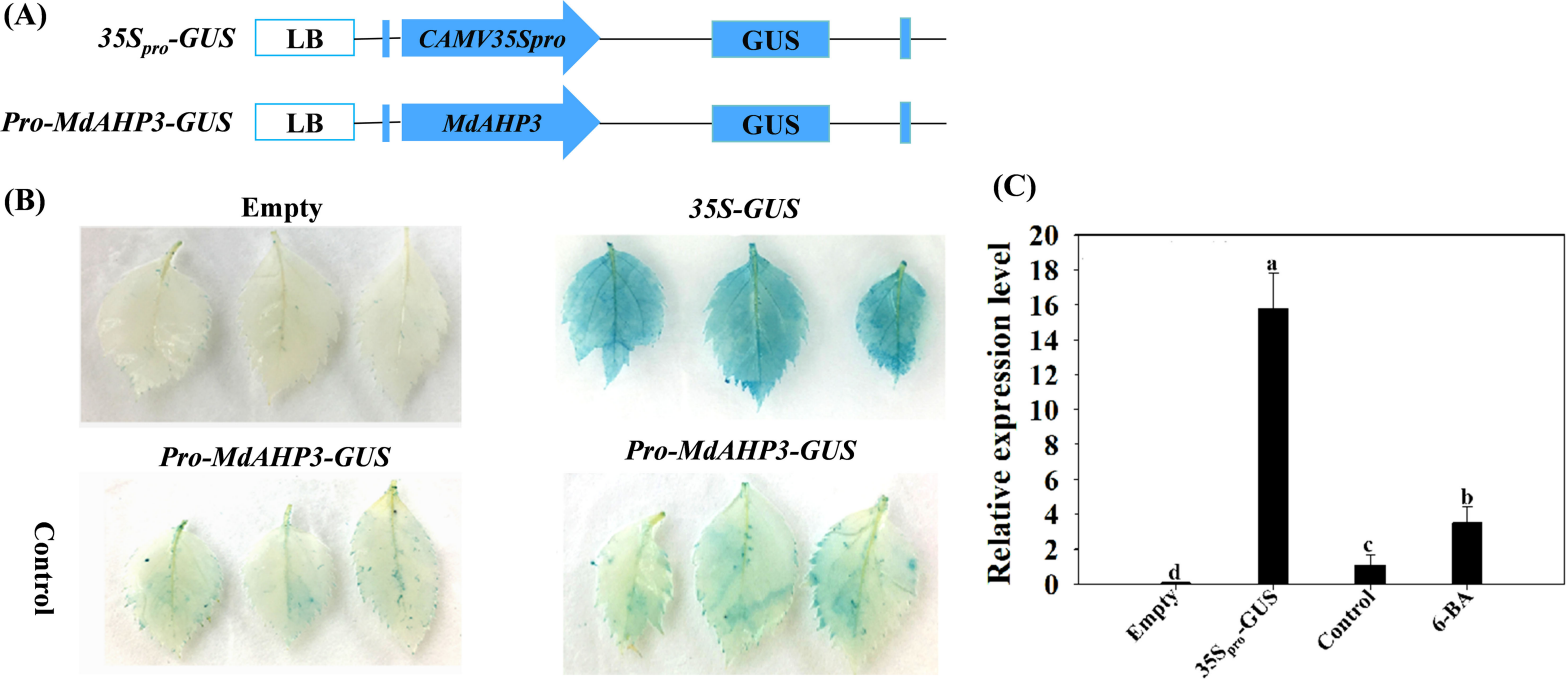
GUS activity of the *MdAHP3* promoter in response to exogenous 6-BA treatment in apple leaves. **(A)** Schematic diagram of the *MdAHP3-GUS* expression vectors. **(B)** *35Spro-GUS*: Empty pBI121 vector (positive control); Empty: No infiltration leaf (blank control). GUS staining images of the *MdAHP3* promoters in response to exogenous 6-BA treatment. Control: Sterile water treatment; 6-BA: Exogenous 100 μmol. L^−1^ IBA treatment. **(C)** Relative expression level of GUS in response to different exogenous hormones. Different letters a–d above the bars indicate significant differences (P < 0.05) among different treatments. Three independent experiments were conducted.

To further determine the response of the MdAHP family members to CTK signaling, the key member *MdAHP3* was selected for GUS staining activity analysis. The recombinant plasmid *pro-MdAHP3-GUS* was transformed into agrobacterium GV3101 and instantly transformed into apple leaves when the leaves had grown for 4 weeks. The results revealed that the *pro-MdAHP3-GUS* staining of apple leaves under 6-BA treatment conditions was significantly higher than that of the control (**Figure 7A**). The GUS activity assay also indicated that *35Spro-GUS* activity was the strongest, while *pro-MdAHP3-GUS* activity under 6-BA treatment was significantly higher than that of the control (**Figure 7B**). The above series of findings suggested that *MdAHP3* could significantly enhance its promoter activity in response to 6-BA signal treatment.

### *MdAHP3* interacts with *MdAHP1* and *MdAHP6* synergistic regulation of AR rormation

**Figure 8:**
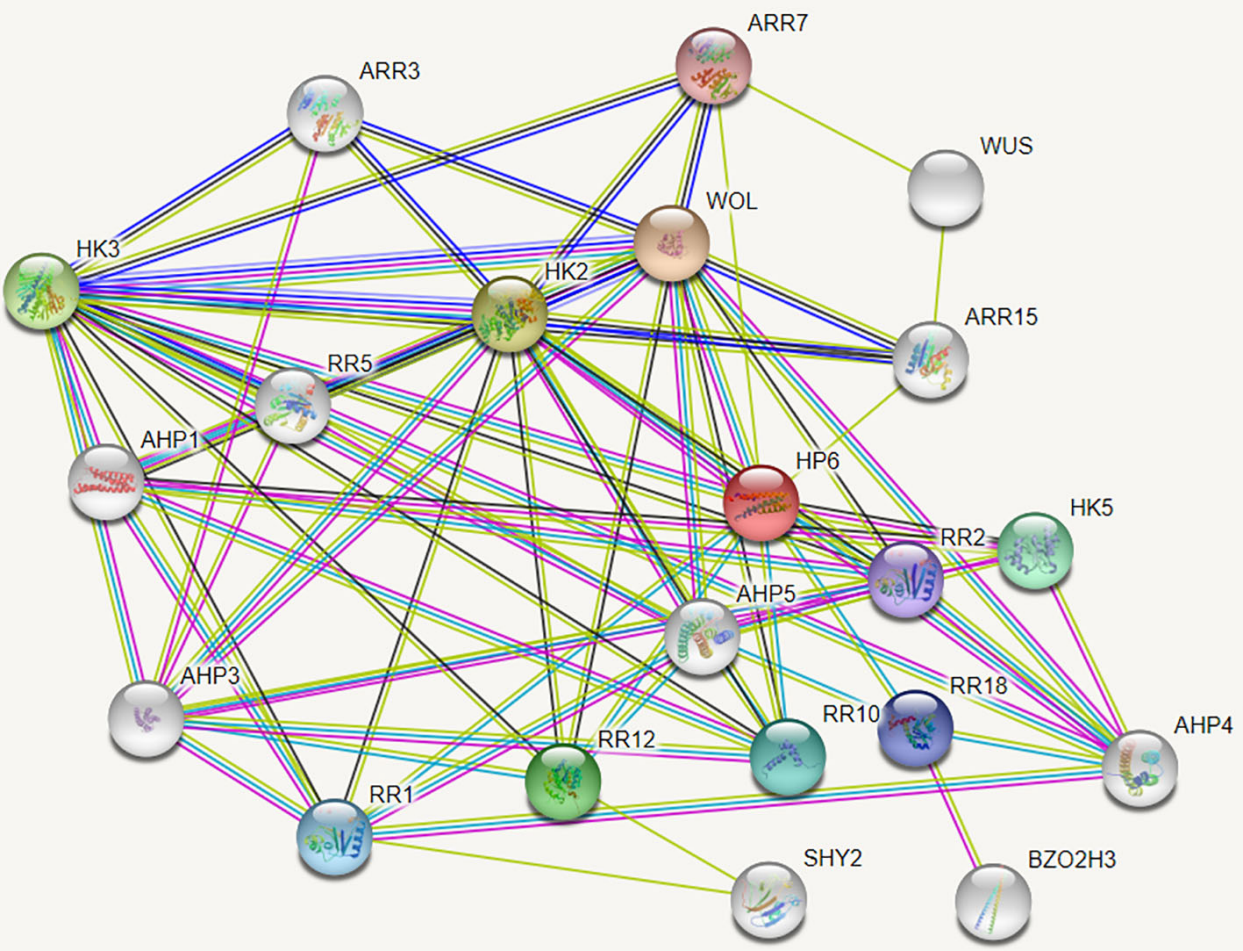
The network of functional connections for MdAHP proteins.

**Figure 9:**
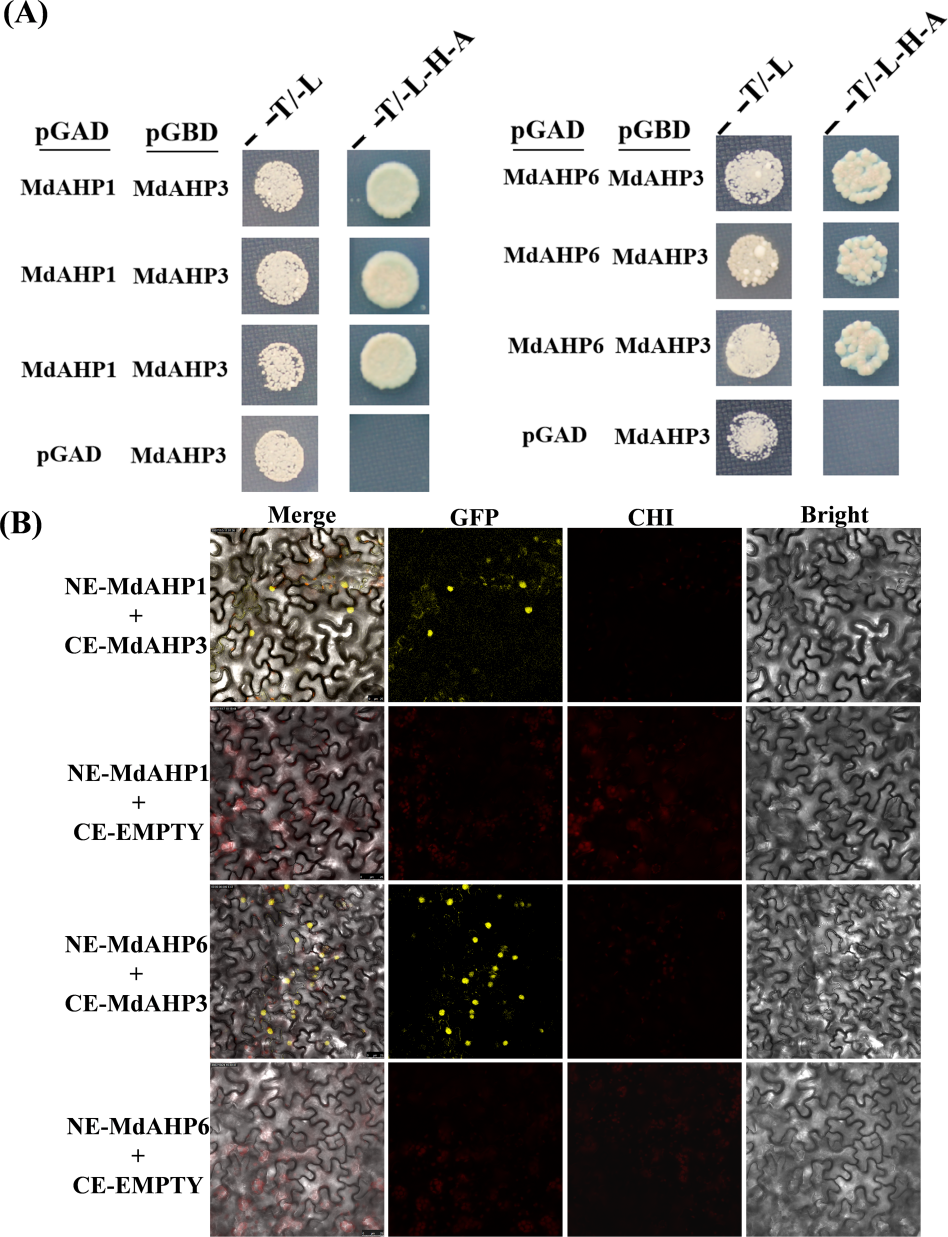
MdAHP3 interacted with MdAHP1 and MdAHP6 *in vitro* and *in vivo*, espectively. **(A)** The empty pGADT7 vector was employed as a control. Transformed yeast cells were cultivated on SD/-Trp/-Leu (T/L) medium or SD/-Trp/-Leu/-His/-Ade (T/L/H/A) medium. **(B)** BiFC analyses were conducted to test the interactions in tobacco leaf epidermal cells. The empty pSPYCE vector served as the negative control. Merge represents the merged images of fluorescence (YFP), chlorophyll autofluorescence, and brightfield images. Bars = 50 μm.

The String protein interaction database was employed to predict the interaction proteins of MdAHP3, and the results are presented in **Figure 8**. These results suggest that MdAHP3 might interact with these transcription factors to carry out regulatory functions. From this, the MdAHP family members MdAHP1 and MdAHP6, which interact with MdAHP3 in mediating AR formation, were selected. Additionally, the interactions between MdAHP1 and MdAHP6 of the MdAHP family and the MdAHP3 protein were demonstrated by yeast two-hybrid and bimolecular fluorescence complementation assays (**Figure 9**).

### Ectopic over-expressions of *MdAHP3* in poplar inhibit AR formation

**Figure 10:**
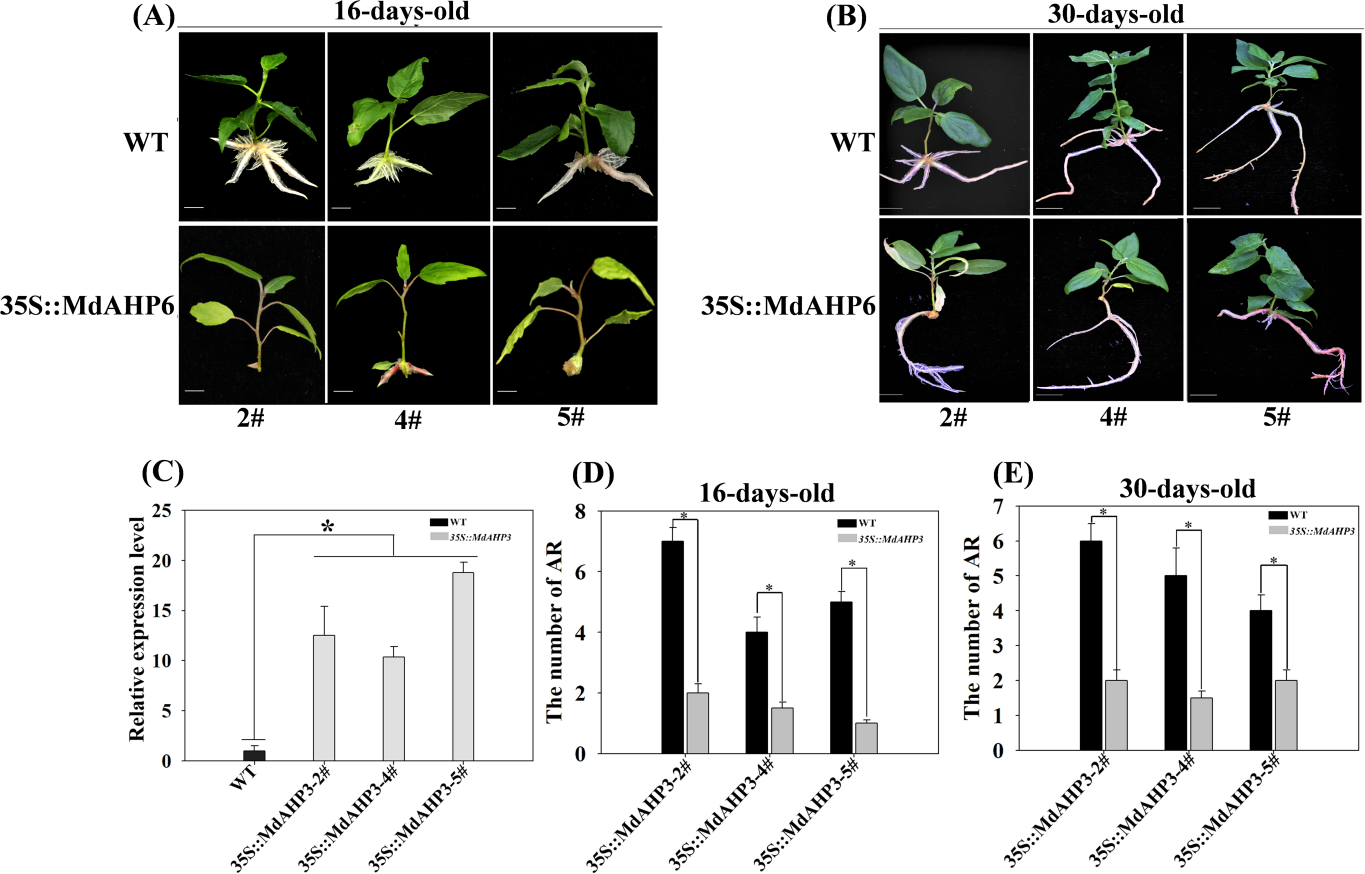
The phenotypes of AR formation in heterologous overexpression *MdAHP3* poplar transgenic plants. **(A)** The AR phenotype of WT and *35S::MdAHP3* transgenic poplars under rooting treatment. Seedlings are grown in one-half strength MS medium with 0.5 mg. L^−1^ IBA treatment under long daylight conditions for approximately 16 days. **(B)** The AR phenotype of WT and *35S::MdAHP3* transgenic poplars under rooting treatment. Seedlings are grown in one-half strength MS medium with 0.5 mg. L^−1^ IBA treatment under long daylight conditions for approximately 30 days. **(C)** The relative expression level of WT and *35S::MdAHP3* transgenic poplars. **(D)** The number of AR in WT and *35S::MdAHP3* transgenic poplars under control and IBA treatments at 16 days. **(E)** The number of AR in WT and *35S::MdAHP3* transgenic poplars under control and IBA treatments at 30 days. Each experiment was completed with three replicates. Asterisks indicate a significant difference (p < 0.05).

To further illustrate the involvement of MdAHP family genes in the regulation of AR formation, *MdAHP3* was ultimately selected as a key member based on the above analysis. We constructed a transgenic plant heterologously over-expressing *35S::MdAHP3* to enhance the expression activity of *MdAHP3* in *poplar*. Three transgenic lines (#2, #4, and #5) were obtained. The phenotypes of *35S::MdAHP3* transgenic plants were identified under rooting treatment conditions (IBA treatment), and the AR phenotypes were analyzed at 16 and 30 days after treatment, with wild-type plants as controls (**Figures 10A, B**). The expression of *MdAHP3* was detected in WT and *35S::MdAHP3* transgenic poplars. The results indicated that the expression level of *MdAHP3* in transgenic poplars was significantly higher than that in WT (**Figure 10C**). Additionally, the number of ARs was counted (**Figures 10D, E**). From the results, it can be seen that *35S::MdAHP3* transgenic poplars inhibited AR formation compared with WT, and showed a significantly reduced number of ARs at 16 and 30 d, respectively (**Figure 10**).

## Discussion

### Identification of Apple *AHP* genes

Apple fruit trees are widely cultivated worldwide. AR formation is an essential step for the mass propagation of apple rootstocks. The formation of ARs is a complex process, affected by various external and internal factors. CTK inhibits plant AR or lateral root production at multiple levels of metabolism, signal transduction, and translocation (Marhavy et al., 2014). We identified 13 MdAHP genes in the apple genome, a number significantly greater than that of AHP genes in *Arabidopsis*. This might reflect that the apple genome, approximately 881 Mb, is much larger than that of *Arabidopsis*, about 12 Mb. The identified MdAHP genes were unevenly distributed on apple chromosomes 2, 3, 4, 8, 9, 11, 12, 13, 14, 15, 16, and 17 (**Table 1**). Multiple sequence alignment revealed that the majority of MdAHP proteins contained a series of conserved domains (**Figure 1**). Promoter fusions of AHP genes showed that *AHP1*, *AHP2*, *AHP3*, and *AHP5* were prominently expressed in embryo sacs, especially in the central cell. Additionally, analyses of *ahp* knockout mutants suggested their redundant function in CTK signalling and plant development (Liu et al., 2017; Keiichi et al., 2010). The results indicated that MdAHP family members have a high degree of homology among them and possible functional redundancy, but the specific functions still need further characterization.

### Phylogenesis, evolution, expansion of *MdAHP* genes

An unrooted neighbor-joining tree was constructed based on the multiple alignment of the AHP protein sequences from *apple* and *Arabidopsis* to explore evolutionary relationships. The analysis divided the AHP proteins into three groups: *MdAHP3*, *MdAHP7*, *MdAHP8*, and *MdAHP12* were clustered and belonged to the Class I group; *MdAHP2*, *MdAHP6*, *MdAHP13*, and *MdAHP9* were clustered and belonged to the Class II group; *MdAHP4*, *MdAHP10*, *MdAHP5*, *MdAHP1*, and *MdAHP11* were clustered and belonged to the Class III group (**Figure 3**). Additionally, MdAHP1, MdAHP6, and MdAHP3 from the three subfamilies have protein interactions (**Figures 8**, **9**), and this result suggests that key members of this gene family are involved in regulating plant development through interactions.

Previous research has shown that gene duplications are important in the evolution of species. Genome-wide duplication events occurred in *apple* about 60 million years ago, resulting in expansion from nine to 17 chromosomes and diversification of some gene families (Velasco et al., 2010). A number of apple gene duplications have been reported, such as the FKBPs (Dong et al., 2018), CIPK (Hai et al., 2018) and HSP families (Yao et al., 2020). In the present study, 11 genes were identified (**Figure 2**). Gene duplications and expansion resulted in MdAHP gene clusters and increased the diversification of MdAHP genes structures and functions.

Genomic comparisons with orthologous genes from well-studied plant species may provide a valuable reference for newly identified genes (Wang et al., 2018, Koonin, 2005). Thus, the functions of MdAHPs were inferred through comparative genomic analyses with the AHP genes from *Arabidopsis*. Three family groups between *Arabidopsis* and *apple* were identified (**Figure 2B**), which suggested that the genes in question may share a common ancestor and their functions have been conserved during evolution. Although many genetic prediction resources are available, additional research is needed to determine the specific function of each gene.

### *MdAHP3* interacted with *MdAHP1* and *MdAHP6* mediating CTK signal transaction

Given the functional diversity of the AHP gene family, all members of the *MdAHP* gene family need to be further investigated in terms of functionality. Analyzing the tissue expression patterns of *MdAHP* genes can provide a preliminary understanding of their potential functions. Analyses of expression patterns in different species and tissues revealed that most members of the *MdAHP* family were highly expressed in leaves, flowers, and fruits, while they were less expressed in roots (**Figure 4**), and the results are consistent with the previous results of AHP inhibiting root development in *Arabidops*is (Hradilová and Brzobohaty, 2007). Additionally, we used ‘M9-T337’ as the material to determine the MdAHP family members in its flowers, fruits, leaves, stems, flower buds, axillary buds, lateral roots, and fibrous roots (**Figure 6**). It is notable that the expression of *MdAHP1* in roots is exceptionally low (**Figure 6**). This might be attributed to the inhibitory effect of this gene in regulating root system development. In the subsequent research, we will also lay emphasis on conducting an in-depth investigation into the expression characteristics and functions of *MdAHP1* in regulating AR formation. In conclusion, the expression of *MdAHP* family members between tissues suggests that the family members may also have different functions in regulating organ development; however, its specific gene functions still need to be verified in further experiments. On the other hand, according to a more accurate *MdAHP3* promoter-driven GUS staining pattern (**Figure 7**), the class I of AHP genes, *AHP3*, was expressed at a low level in flower, leaf, and stem tissues and at a high level in fruit, and moderately in flower buds, axillary buds, lateral roots, and fibrous roots; moreover, cytokines have a significant effect on the expression profile of *AHP3* (Hai et al., 2005; Zhao et al., 2021). Therefore, we inferred that the developmental stage, sampling method, and species specificity may affect the experimental results. Previous research indicated that auxin-cytokines homeostasis in the AR formation of rose cuttings is affected by their nodal position in the stock plant (Otiende et al., 2021).The balance between CTK and auxin is also a major determinant of the cell fate reorganization mechanism in plant tissues (Rasmussen et al., 2017). CTK signaling and perception are necessary for plant root development (Mahonen et al., 2006). while auxin is also involved in regulating AR induction mechanisms (Sukumar, 2010).

From the above background and the results of the present study, it is hypothesized that *MdAHP3*, as a representative member of the MdAHP family, may mediate CTK signaling to regulate AR formation; however, the specific function still needs further validation. In the current study, in combination with exogenous 6-BA and the CTK inhibitor Lov treatment, their expression characteristics were determined during the critical stage of AR development. *MdAHP3* was significantly up-regulated by 6-BA treatment during the induction and initiation stages (3 and 7 d) of AR formation, which also suggested that *MdAHP3*, as a key member, might regulate AR formation (**Figure 5**, **Supplementary Figure 1**). The results were consistent with the report that *AHP3* in *Arabidopsis* is involved in the regulation of root development by cytokinin signaling (Suzuki et al., 2000; Yadav et al., 2022; Wang et al., 2018). Additionally, the promoter activity of *MdAHP3* was significantly enhanced by 6-BA induction (**Figure 7**). From the above background and the results of the present study, it is hypothesized that *MdAHP3*, as a representative member of the MdAHP family, may mediate CTK signaling to regulate AR formation; however, the specific function still needs further validation.

### MdAHP3, as a key number of the MdAHPs family, functions as a suppressor to regulate AR formation

From the foregoing results, we have identified *MdAHP3* as a key member of the MdAHP family in apple, which might exert its functions in regulating AR formation (**Figures 4**–**7**). Functional redundancy exists among different gene families, where no single member acts alone (Wightman, 2003; Pérez-Pérez et al., 2013). the same is presumed for the MdAHP family. Therefore, with the identified MdAHP3 as the core, the interaction proteins (MdAHP1 and MdAHP6) were predicted and validated through a combination of yeast two-hybrid and bi-molecular fluorescence complementation assays (**Figure 9**). The findings in apple were in line with previous research that the sensitivity to exogenous cytokinin was not obviously influenced for each ahp single mutant, but was significantly reduced in the *ahp1, 2, 3* triple mutant. Specifically, *ahp1*, *ahp2*, *ahp3*, *ahp4*, and *ahp5* did not respond to cytokinin and were accompanied by severe developmental defects, indicating that AHPs act redundantly as positive regulators in the two-component signaling pathway (Schaller et al., 2008; Deng et al., 2010; Hutchison et al., 2006). AHPs have been demonstrated to serve as bridges for multi-step phosphorelays between AHKs and ARRs, consistent with their function as mediators of the CTK pathway (Mira-Rodado et al., 2012). Additionally, to further verify that MdAHP family members mediate CTK signaling to regulate AR formation in apple rootstock, it was found that heterologous overexpression of *MdAHP3* in poplar inhibits AR formation through phenotype identification (**Figure 10**). Previous studies suggested that the *ahp6* mutant could partially restrain wol phenotypes, and the activity of *AHP6* is repressed by cytokinin (Mahonen et al., 2006; Sofia et al., 2013), indicating its role as a negative regulator of cytokinin response. Furthermore, a low level of CTK is necessary for root primordium formation, while a high level of CTK strongly suppresses root formation (Hou and Wang, 2010). Integrating previous reports and the results of the present study, we hypothesize that MdAHP3 interacts with proteins (MdAHP1 and MdAHP6) to mediate CTK signaling, which in turn governs AR formation. Overall, the current study systematically identified the physiological and biochemical characteristics of the MdAHP family members in apples and screened the key members that might be implicated in AR formation. It has laid the foundation for the subsequent research on the molecular mechanism of AR formation in apple rootstocks; nevertheless, the specific regulatory mechanism still requires further investigation.

## Conclusion

**Figure 11:**
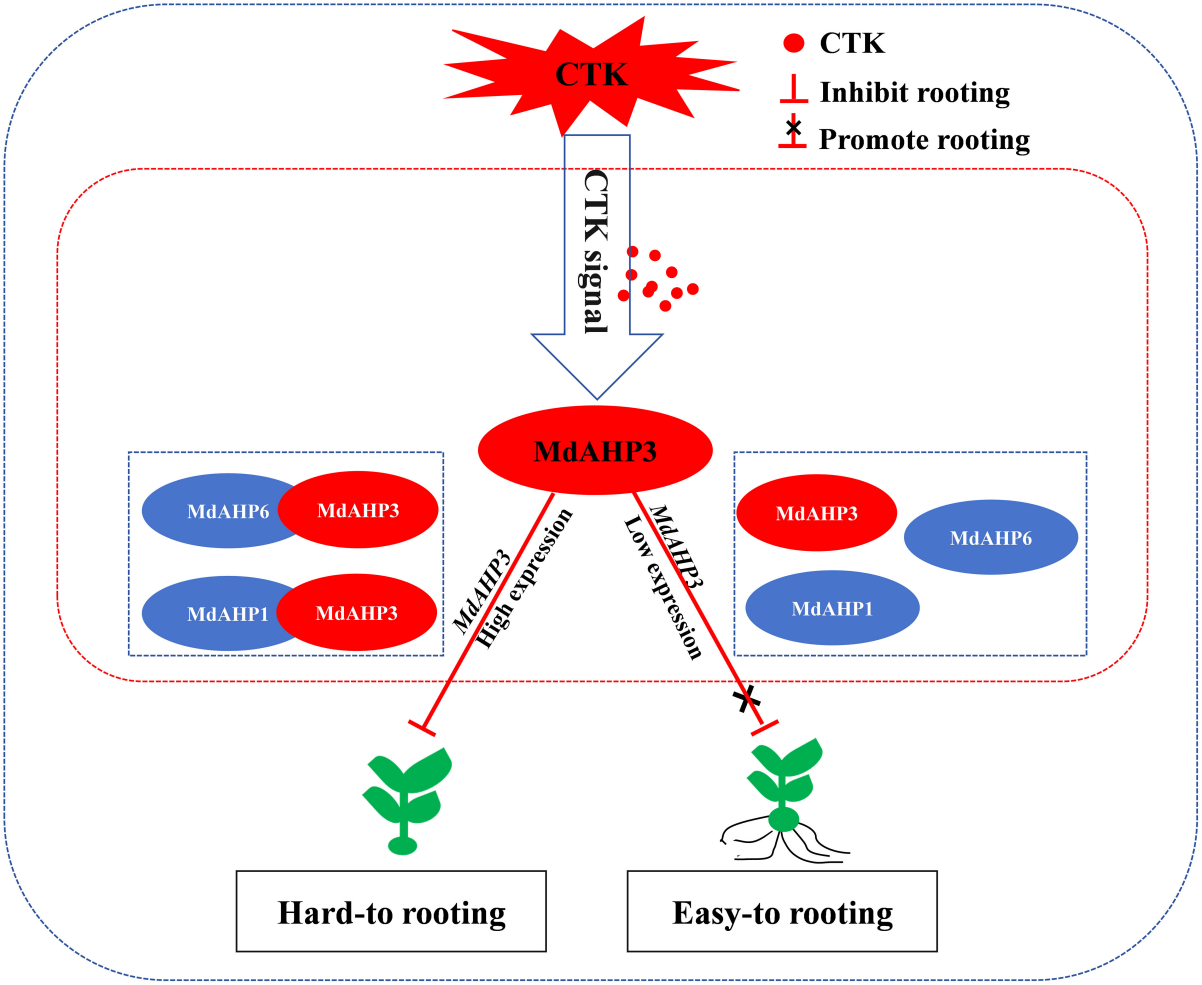
The working model of *MdAHP3*, as a key family member of MdAHP family gene, interacting with *MdAHP1/MdAHP6* to mediate CTK signal regulation of AR formation in apple. Note: In difficult-to-root apple rootstocks, *MdAHP3* is induced by high-concentration CTK signals and its expression is up-regulated, thereby inhibiting the occurrence of AR primordia at the stem base of seedlings. In easy-to-root apple rootstocks, low-concentration CTK signals inhibit the high expression of *MdAHP3*, thereby blocking the inhibitory effect of CTK signals on AR formation and promoting the occurrence of AR primordia at the stem base of seedlings.

AR formation constitutes a bottleneck for mass propagation in apple rootstocks. Cytokinin, as a major plant hormone, mediates root development, and AHP is a key member in CTK signal transduction. In this research, a total of 13 AHP genes were identified in *apple* and phylogenetically categorized into three clusters. Additionally, the expression pattern of MdAHPs during the critical stages of AR formation was analyzed in ‘M9-T337’ stem cuttings. When combined with exogenous 6-BA and cytokinin inhibitor (Lov) treatments, the results demonstrated that the expression pattern was significantly modified by exogenous CTK signaling at the stages of AR formation. There were also notable differences in the expression of MdAHP family members among different tissues. Furthermore, the existence of protein interactions of MdAHP3 with MdAHP1 and MdAHP6 was verified *in vitro* and *in vivo* (**Figures 9**, **11**). Integrated with family identification and gene expression analysis, the preliminary screening suggested that *MdAHP3* could act as a key member in mediating CTK signaling. The promoter activity was significantly enhanced by 6-BA induction, and heterologous overexpression of *35S::MdAHP3* in transgenic *poplar* inhibited AR formation (**Figure 11**). To our knowledge, this study represents the first systematic and in-depth analysis of apple AHP genes. The data offer valuable information for the future functional characterization and regulation mechanism of MdAHPs in *apple*.

## Data Availability

The datasets presented in this study can be found in online repositories. The names of the repository/repositories and accession number(s) can be found in the article/**Supplementary Material.**

## Supplementary material

Supplementary Figure 1 Heat map diagram of the MdAHPs family genes.
