# Supplementary figures and images for: Identification of the AHP family reveals their critical response to cytokinin regulation during adventitious root formation in apple rootstock
Source: Front Plant Sci. 2025 Jan 15;15:1511713. doi: 10.3389/fpls.2024.1511713 (PMC11776435; doi:10.3389/fpls.2024.1511713)

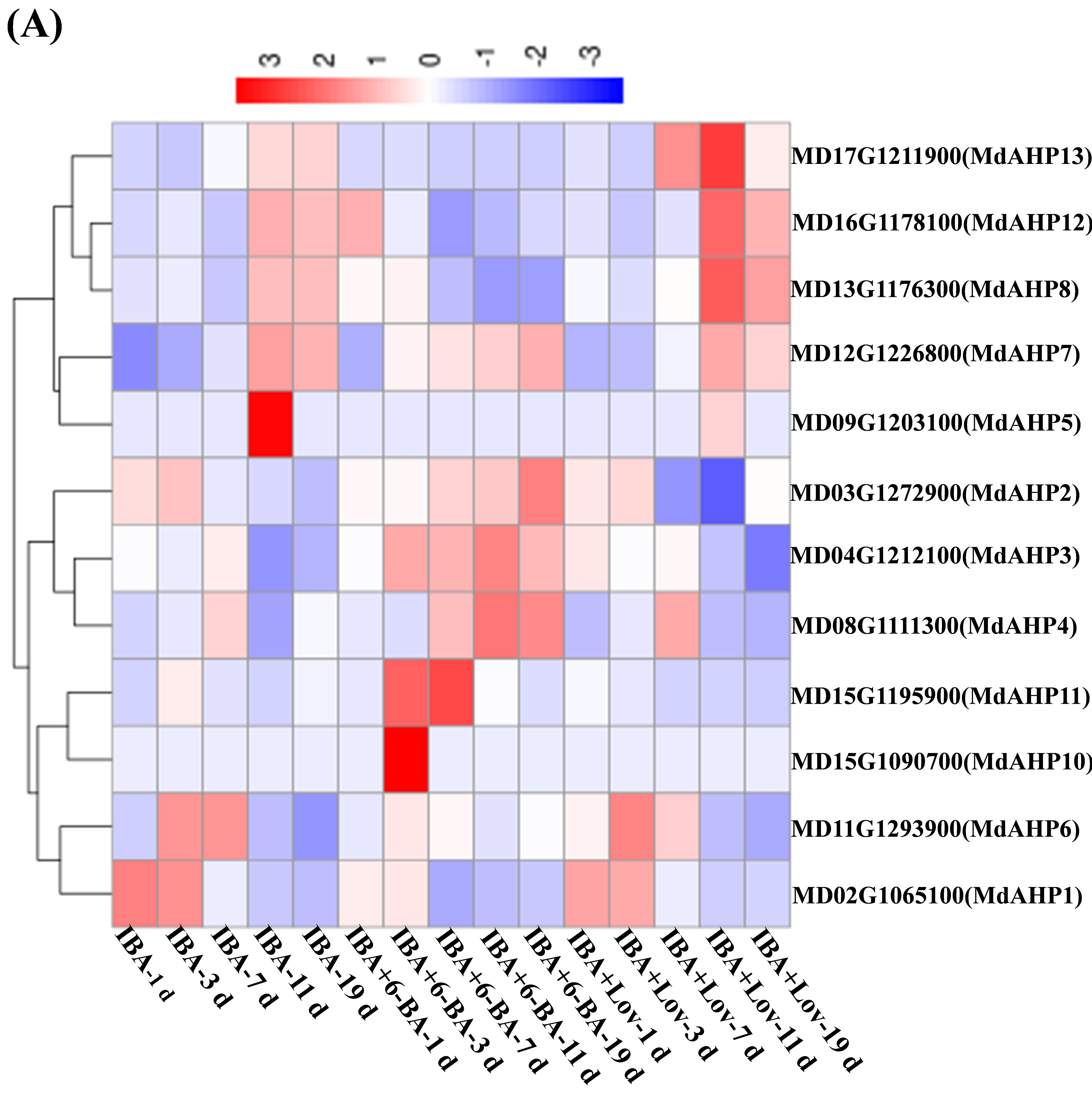

Supplement: Supplementary Figure 1 — Heat map diagram of the MdAHPs family genes. [file Image1.tif]
